# Fibroblast activation protein inhibitor-PET/CT in lung cancer: a prospective single-center study with histopathological correlation of fibroblast activation protein expression

**DOI:** 10.1007/s10147-026-03047-6

**Published:** 2026-06-02

**Authors:** Takashi Hiroshima, Toru Kimura, Tadashi Watabe, Takahiro Matsui, Akiisa Omura, Yusuke Sugiura, Hideki Nagata, Kenji Kimura, Eriko Fukui, Takashi Kanou, Naoko Ose, Mitsuaki Tatsumi, Frederik L. Giesel, Eiichi Morii, Yasushi Shintani

**Affiliations:** 1 Department of General Thoracic Surgery, The University of Osaka Graduate School of Medicine, Suita, Japan; 2 Division of Clinical Translation, Department of General Thoracic Surgery Institute for Radiation Sciences, The University of Osaka Graduate School of Medicine, Suita, Japan; 3 Department of Pathology, The University of Osaka Graduate School of Medicine, Suita, Japan; 4 Department of Diagnostic Radiology, Faculty of Medicine, Juntendo University, Tokyo, Japan; 5 Department of Nuclear Medicine, University Hospital Düsseldorf, Heinrich Heine University, Düsseldorf, Germany

**Keywords:** Lung cancer, [^18^F]FAPI-74 PET, Fibroblast activation protein, Cancer-associated fibroblasts, Lymph node metastasis

## Abstract

**Background:**

Recent studies have demonstrated the utility of fibroblast activation protein inhibitor (FAPI)-positron emission tomography (PET) for cancer diagnosis. We investigated the diagnostic capability of [^18^F]FAPI-74 PET in patients with lung cancer eligible for surgery.

**Methods:**

This study included 30 patients (June 2022 to February 2024) who underwent [^18^F]FAPI-74 PET followed by lung resection at our institution and were pathologically diagnosed with primary lung cancer. The histological types included 24 adenocarcinomas, four squamous cell carcinomas, and two small cell carcinomas or combined small cell carcinomas and adenocarcinomas. Fibroblast activation protein (FAP) expression was evaluated in surgically resected specimens of primary tumors and lymph nodes using immunohistochemical staining.

**Results:**

All 30 primary lung lesions showed uptake on [^18^F]FAPI-74 PET (median maximum standardized uptake value [SUVmax] = 4.8; range: 1.2–15.5). A positive correlation was observed between the SUVmax of [^18^F]FAPI-74 PET and FAP expression by immunohistochemical staining (FAP-positive area, *p* = 0.0083; H-score, *p* = 0.0076). The sensitivity and specificity of [^18^F]FAPI-74 PET for the diagnosis of lymph node metastases were 75.0% and 88.5%, respectively. In all six cases of significant [^18^F]FAPI-74 uptake in lymph nodes, FAP expression was confirmed in the lymph node tissue by immunohistochemical staining. Three patients demonstrated lymph node uptake on [^18^F]FAPI-74 PET due to preoperative chemoradiation therapy or reactive lymph node enlargement/uptake without any malignant findings.

**Conclusions:**

In resectable early-stage lung cancer, [^18^F]FAPI-74 PET correlated with histological FAP expression and proved useful for detecting lymph node metastases, although caution is warranted when interpreting the findings after preoperative therapy.

## Introduction

Lung cancer is the leading cause of cancer-related mortality worldwide, accounting for approximately 1.8 million deaths annually [1]. Despite advances in diagnostic imaging and therapeutic strategies, prognosis remains poor, underscoring the need for improved approaches for early detection and precise disease characterization. Accumulating evidence indicates that cancer progression and response to therapy are not solely determined by the tumor cells themselves but are strongly influenced by the surrounding tumor microenvironment. Among stromal components, cancer-associated fibroblasts (CAFs) represent a major cellular subset that plays a pivotal role in tumor initiation, progression, and therapy resistance [2].

Fibroblast activation protein (FAP), a type II integral membrane serine protease, is preferentially expressed on CAFs but has limited expression in normal adult tissues. Consequently, FAP has emerged as an important biomarker for identifying CAFs and is a promising target for imaging and therapeutic applications [3]. Accurate staging of lung cancer is crucial for determining optimal treatment strategies and predicting clinical outcomes [4]. Conventional fluorodeoxyglucose positron emission tomography (FDG-PET) has been used to evaluate the pulmonary and mediastinal lymph nodes in patients with lung cancer [5, 6]. However, FDG uptake is neither tumor-specific nor consistent, and its diagnostic accuracy can be compromised by false-positive findings due to inflammatory uptake and low glucose metabolism in adenocarcinoma [7]. In recent years, the development of fibroblast activation protein inhibitors (FAPI) labeled with positron emission tomography (PET) radionuclides has provided a new imaging tool capable of visualizing cancer and metastatic lesions with high sensitivity and specificity. Several clinical reports have demonstrated that FAPI-PET outperforms FDG-PET in various malignancies by offering superior tumor-to-background contrast and improved primary and metastatic lesion detection [8]. These findings suggest that FAPI may provide precise staging and treatment planning for lung cancer.

In this study, we aimed to evaluate the diagnostic performance of [^18^F]FAPI-74 PET in patients with lung cancer in the preoperative setting. Specifically, we compared the abilities of [^18^F]FAPI-74 PET and FDG-PET to detect primary lesions and nodal involvement, thereby assessing the potential clinical utility of [^18^F]FAPI-74 PET in improving surgical decision-making.

## Patients and methods

### Patient selection

Between June 2022 and February 2024, 41 patients underwent [^18^F]FAPI-74 PET at our hospital as part of a prospective clinical study. Of these, 11 were excluded because the pathological diagnosis was not primary lung carcinoma; excluded cases included thymic epithelial tumors (n = 7), benign lesions (n = 3), and a metastatic lung tumor (n = 1). Ultimately, 30 patients with surgically resected, pathologically confirmed primary lung carcinoma were included in the analysis. The clinical and demographic characteristics of the study cohort are summarized in Table 1.

**Table 1:** Patient Characteristics

Variable	All patients (n = 30)
Age (mean, years)	73.5 ± 7.3
Sex (n)	
Male	22
Female	8
Surgical procedure (n)	
Lobectomy	21
Segmentectomy	7
Wedge resection	2
Histological type (n)	
Ad	24
Sq	4
SCLC or C-SCLC	2
Induction therapy (n)	2
Tumor size (mean, cm)	2.3 ± 1.0
Pathologically proven lymph node metastasis (n)	4
Pathological stage (n)	
0	3^a^
Ⅰ	19
Ⅱ	6
Ⅲ	1^a^
Ⅳ	1

^a^One case including preoperative chemoradiotherapy

Ad, adenocarcinoma; Sq, squamous cell carcinoma; SCLC, small cell lung carcinoma; C-SCLC, combined small cell lung carcinoma

Histopathological evaluation identified 24 cases of adenocarcinoma, four cases of squamous cell carcinoma, one case of combined small cell carcinoma and adenocarcinoma, and one case of small cell carcinoma. The pathological stages were as follows: stage 0 (adenocarcinoma in situ), two cases; stage I, 19 cases; stage II, six cases; stage III, one case; stage IV, one case; and one case with no residual tumor. We included seven cases from our previous study [9] and conducted additional analyses, including correlations with FAP expression by immunohistochemistry and comparisons based on histological subtype and stage. The study protocol was reviewed and approved by the Institutional Review Board of the University of Osaka Hospital (approval number: 21472–4) and was conducted in accordance with the Declaration of Helsinki (2024). Written informed consent was obtained from all participants before enrollment.

### PET/Computed tomography scanning and image analysis

PET/computed tomography (CT) was performed 60 min after the intravenous administration of [^18^F]FAPI-74 (3.7 MBq/kg) [9]. All scans were acquired using the Biograph Vision 600 PET/CT system (Siemens Healthineers, Erlangen, Germany). PET images were reconstructed using an ordered subset expectation maximization algorithm (three iterations, five subsets) with a 3-mm Gaussian filter. Attenuation correction was performed using low-dose unenhanced CT (effective dose, 50 mAs).

Quantitative image analysis was performed using the Syngo.via software (Siemens Healthineers). Volumes of interest (VOIs) were manually placed on the primary lung tumor, metastatic lymph nodes, and distant lesions to obtain the maximum standardized uptake values (SUVmax). In 23 of the 30 patients, a comparative [^18^F]FDG-PET scan was available within 12 weeks (median interval: 5.1 weeks), and [^18^F]FDG-PET scans were obtained for routine clinical management.

### Immunohistochemical staining

Formalin-fixed paraffin-embedded tumor tissues were sectioned at a thickness of 4 μm. Sections were deparaffinized with xylene and rehydrated using a graded alcohol series. To retrieve the antibody-specific antigens, the slides were treated with 10 mM sodium citrate buffer containing 0.05% Tween for 20 min at 100 °C. Endogenous peroxidase activity was blocked using 3% hydrogen peroxide, followed by incubation with 10% goat serum and 1% bovine serum albumin to reduce nonspecific binding. Tissue slides were stained with rabbit anti-human FAP monoclonal IgG (1:250; No. ab207178). The slides were rinsed with phosphate-buffered saline (PBS), and a secondary goat anti-rabbit IgG antibody (horseradish peroxidase polymer) was applied for 60 min at room temperature. After rinsing with PBS, the slides were incubated for 60 s with chromogen 3,3′-diaminobenzidine (DAB) and counterstained with hematoxylin.

FAP expression in the pulmonary lesions was quantified using two complementary methods. First, a quantitative image analysis was performed. Four representative sections from each tumor slide were imaged using an all-in-one fluorescence imaging system (BZ-X700; KEYENCE, Osaka, Japan). The FAP-positive areas in the four sections of each tumor were analyzed using ImageJ Fiji [10] and averaged. Next, each slide was semi-quantitatively scored from 0 (negative) to 3 (strong) by a certified pathologist (T.M.). As previously reported [11], the H-scores were calculated using the following formula: H-score = [0 × (% cells of score 0) + 1 × (% cells of score 1) + 2 × (% cells of score 2) + 3 × (% cells of score 3)]. The presence or absence of FAP expression in lymph node sections was determined by a certified pathologist (T.M.).

### Data analyses

The diagnostic performance of [^18^F]FAPI-74 PET for primary tumors and lymph node metastases was evaluated using pathological findings as the reference standard. SUVmax values were compared according to tumor histological type and pathological stage. For patients who also underwent [^18^F]FDG-PET, a direct comparison with [^18^F]FAPI-74 PET was performed. The correlation between FAP expression, as determined by immunohistochemistry, and SUVmax was also analyzed.

### Statistical analyses

The relationships between continuous variables were assessed using Pearson’s correlation coefficients. For comparisons of medians between groups, either the Wilcoxon rank-sum test or the Wilcoxon signed-rank test was used, as appropriate. Statistical analyses were performed using JMP® statistical software (SAS Institute Inc., Cary, NC, USA). Differences were considered statistically significant at *p* < 0.05.

## Results

**Fig. 1:**
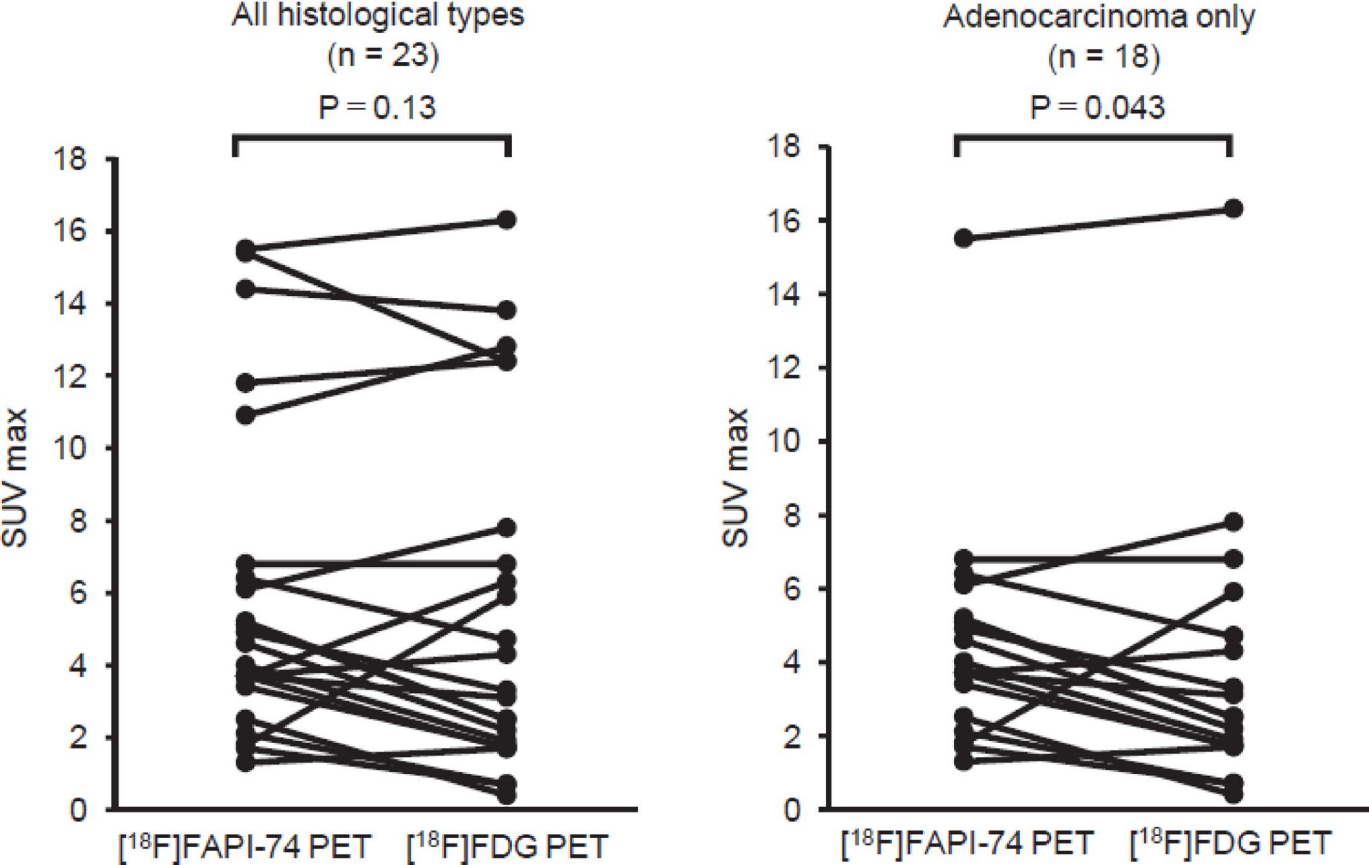
Comparison of SUVmax values between [^18^F]FAPI₋74 PET and [^18^F]FDG PET in pulmonary lesions. The left panel shows SUVmax comparisons in all 23 cases, in which the median SUVmax values were 4.6 for [^18^F]FAPI-74 PET and 3.3 for [^18^F]FDG PET, with no statistically significant difference. The right panel shows comparisons in 18 adenocarcinoma cases, where the median SUVmax values were 3.85 for [^18^F]FAPI-74 PET and 2.5 for [^18^F]FDG PET, demonstrating significantly higher uptake on [^18^F]FAPI-74 PET (*p* = 0.043). SUVmax, maximum standardized uptake value; FAPI, fibroblast activation protein inhibitor; PET, positron emission tomography; FDG, fluorodeoxyglucose

**Fig. 2:**
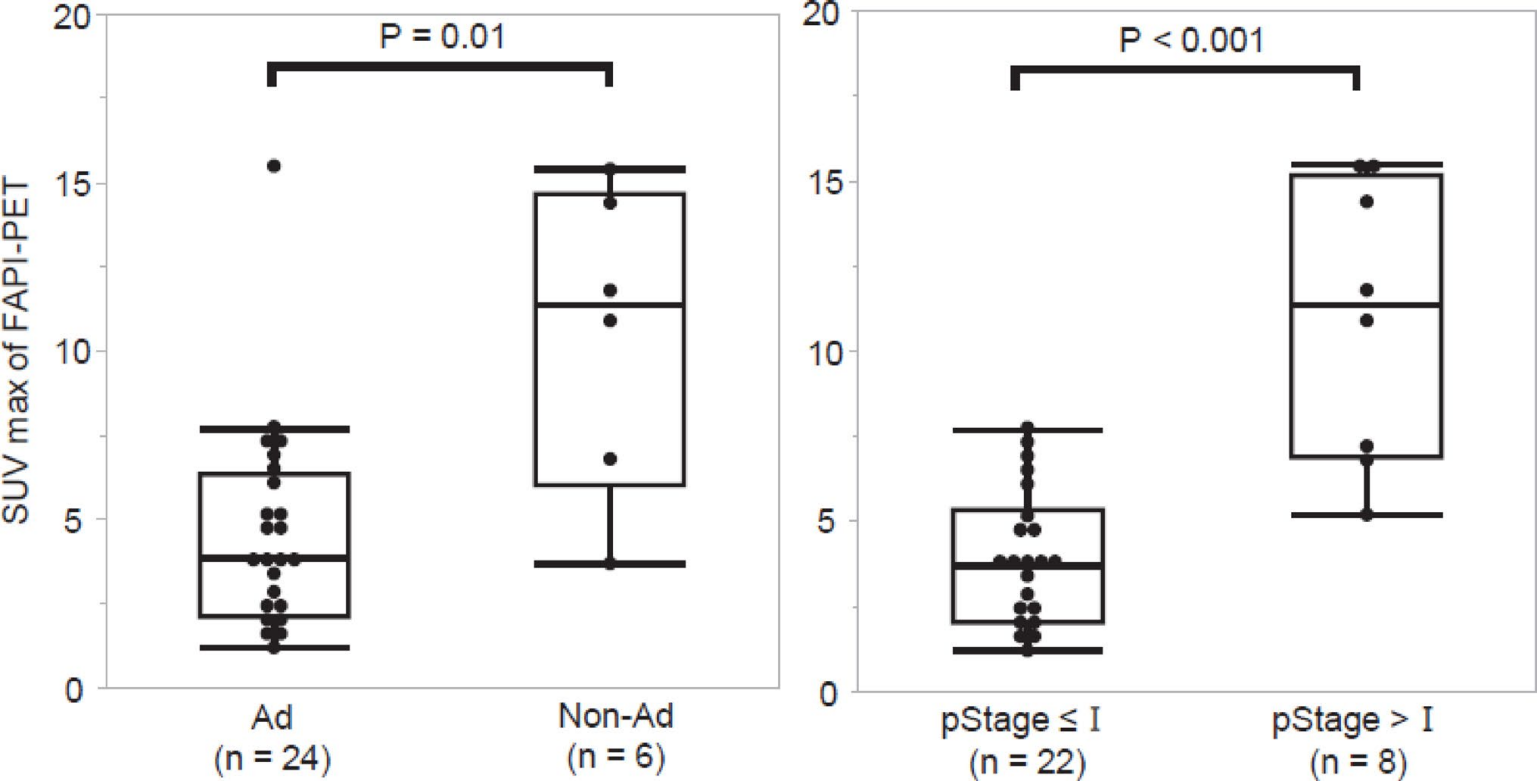
Analysis of SUVmax values of the primary lesion on [^18^F]FAPI-74 PET. Non-adenocarcinoma cases and advanced cases showed high uptake of [^18^F]FAPI-74 PET. SUVmax, maximum standardized uptake value; FAPI, fibroblast activation protein inhibitor; PET, positron emission tomography; Ad, adenocarcinoma

No adverse events attributable to the administration of [^18^F]FAPI-74 were observed in any of the patients who underwent the examination. In the pulmonary nodules of all 30 cases, [^18^F]FAPI-74 PET uptake was detected (median SUVmax = 4.8; range, 1.2–15.5). Among 23 patients who underwent [^18^F]FDG PET during the same period, the SUVmax of the primary tumors was compared. During the interval between FDG-PET and FAPI-PET, patients showed no disease progression that necessitated reconsidering the treatment strategy. In the overall cohort (n = 23), no significant difference was observed (median difference = 1.30, *p* = 0.13); however, in the analysis limited to adenocarcinoma cases (n = 18), [^18^F]FAPI-74 PET demonstrated significantly higher uptake (median difference = 1.35, *p* = 0.043), as shown in Fig. 1. The results of the stratified analysis of SUVmax values on [^18^F]FAPI-74 PET in primary lesions are shown in Fig. 2. Non-adenocarcinoma cases (n = 6) showed higher uptake than adenocarcinoma cases (n = 24) (median SUVmax 11.35 vs. 3.85; *p* = 0.01), and pStage > II cases (n = 8) showed higher uptake than pStage ≤ I cases (n = 22) (median SUVmax 11.35 vs. 3.70; *p* < 0.001).

The sensitivity and specificity of [^18^F]FAPI-74 PET for the diagnosis of lymph node metastasis were 75% (3/4) and 88.5% (23/26), respectively. In contrast, the sensitivity and specificity of [^18^F]FDG PET for diagnosing lymph node metastasis were 50% (2/4) and 84.2% (16/19), respectively.

**Fig. 3:**
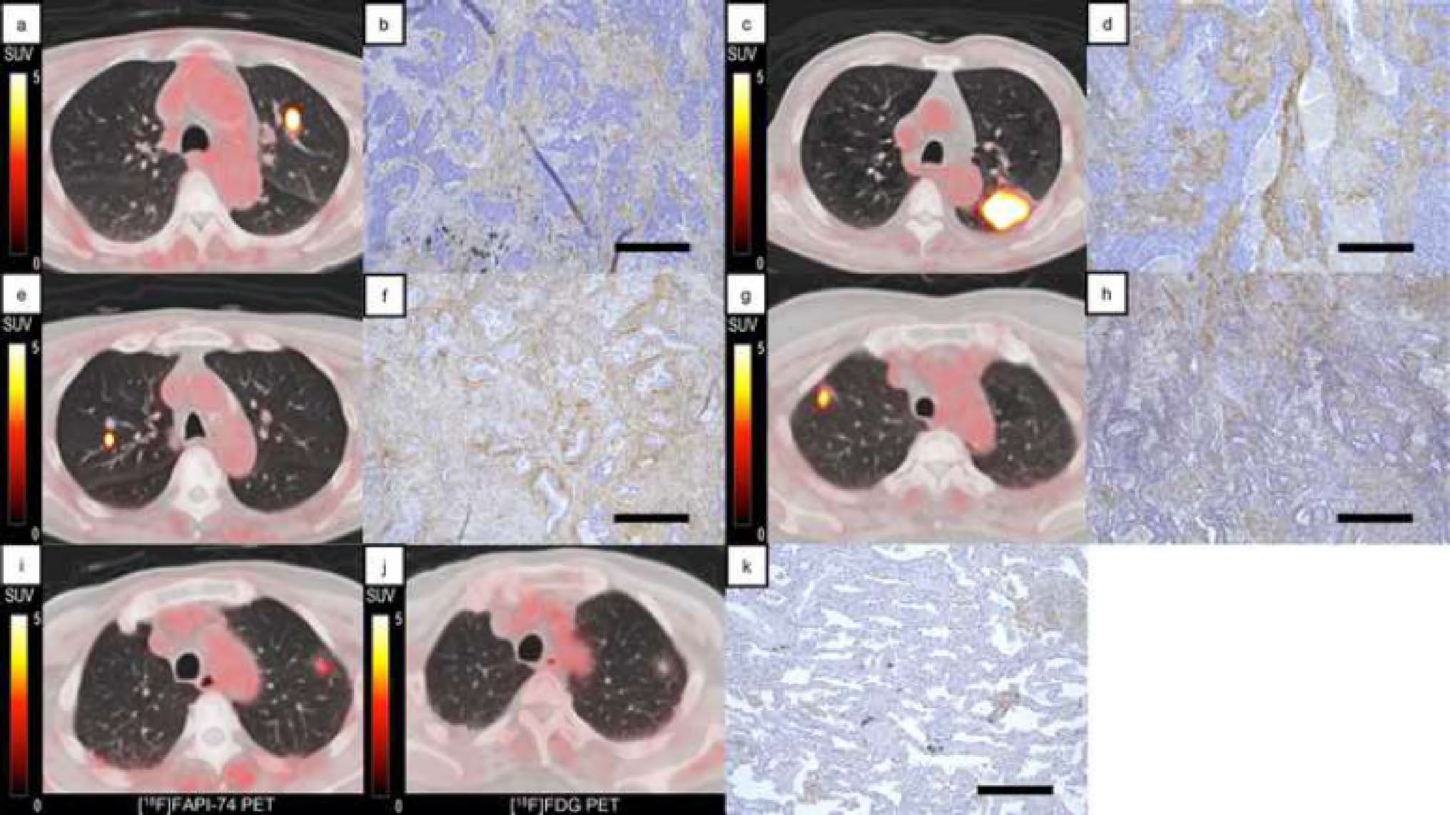
Positron emission tomography (PET)/computed tomography (CT) imaging and fibroblast activation protein (FAP) immunohistochemical staining in pulmonary lesions. **a** and **b** Small cell carcinoma component in combined small cell lung carcinoma. **c** and **d** Squamous cell carcinoma. **e** and **f** Acinar-predominant adenocarcinoma. **g** and **h** Papillary-predominant adenocarcinoma. **i**-**k** Lepidic-predominant adenocarcinoma. Uptake is shown in the pulmonary nodules, during [^18^F] fibroblast activation protein inhibitor (FAPI)-74 PET [**a** maximum standardized uptake value (SUVmax) = 11.8; **c** SUVmax = 10.9; **e** SUVmax = 7.7; **g** SUVmax = 5.1; **i** SUVmax = 2.5]. In the lepidic-predominant case, panel **j** shows fluorodeoxyglucose-PET without significant uptake. FAP expression was confirmed by immunohistochemical staining (**b**, **d**, **f**, **h** and **k**; Scale bar = 300 µm)

**Fig. 4:**
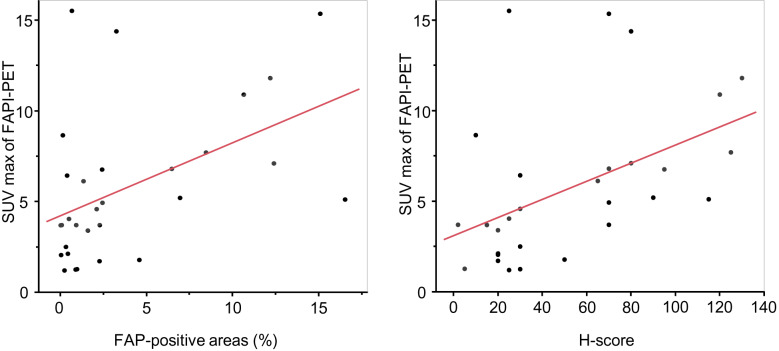
Relationship between the SUVmax of [^18^F]FAPI-74 PET and FAP-positive areas (%) **a** Relationship between the SUVmax of [^18^F]FAPI-74 PET and H-score **b** SUVmax, maximum standardized uptake value; FAPI, fibroblast activation protein inhibitor; PET, positron emission tomography; FAP, fibroblast activation protein

FAP expression was confirmed using immunohistochemistry in all 30 primary lesions. Figure 3 shows the representative FAP immunohistochemistry and [^18^F]FAPI-74 PET images for each histological type. Figures 3i–k show a predominantly lepidic adenocarcinoma case, in which FAP immunohistochemistry stain demonstrated weak FAP expression (Fig. 3k), [^18^F]FAPI-74 PET showed mild uptake with an SUVmax of 2.5 (Fig. 3i), and [^18^F]FDG PET demonstrated no significant uptake (Fig. 3j). The FAP expression was quantified using two approaches: FAP-positive areas (%) and H-scores. As shown in Fig. 4, both measures demonstrated significant correlations with SUVmax on [^18^F]FAPI-74 PET (FAP-positive area (%): r = 0.47, *p* = 0.0083; H-score: r = 0.46, *p* = 0.010).

**Fig. 5:**
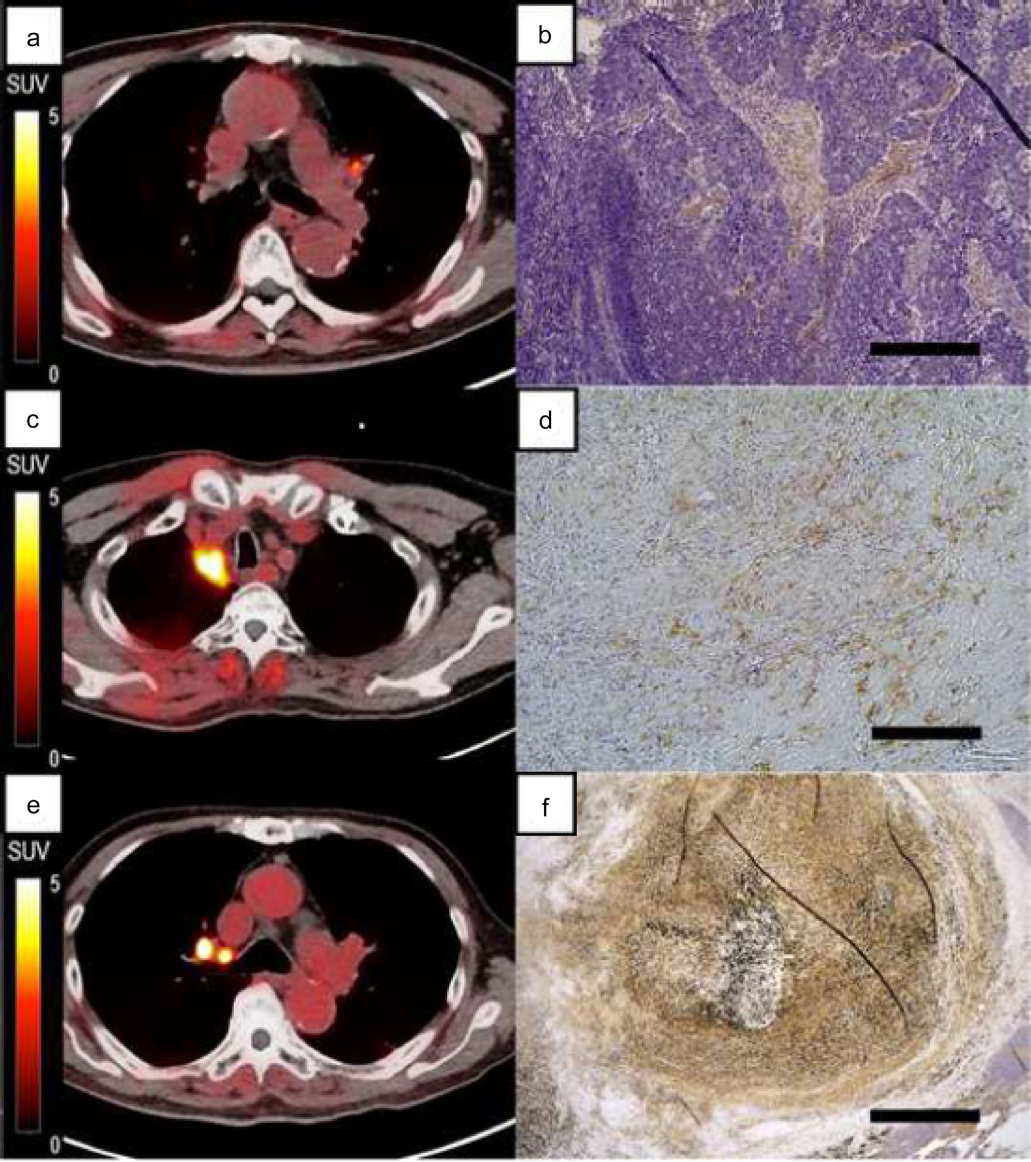
[^18^F]FAPI-74 PET/CT images and FAP immunohistochemistry in representative cases with significant lymph node uptake. **a** and **b** A 77 year-old man with combined small cell carcinoma and adenocarcinoma (pStage IIB). [^18^F]FAPI-74 PET demonstrated focal uptake in a hilar lymph node (SUVmax 3.5) (**a)** FAP-IHC confirmed FAP expression in fibroblasts adjacent to tumor cell nests within the lymph node (**b**). **c** and **d** A 61 year-old man with adenocarcinoma and mediastinal lymph node metastasis who underwent preoperative chemoradiation therapy. [^18^F]FAPI-74 PET obtained after completion of chemoradiation before surgery showed uptake in a mediastinal lymph node (SUVmax 7.3) **c**. Pathology revealed only post-treatment scar tissue without residual malignancy, with FAP expression in the fibrotic area (**d**). **e** and **f** A 79 year-old man with adenocarcinoma (pStage IA) showing uptake in multiple hilar lymph nodes on [^18^F]FAPI-74 PET (SUVmax 11.2) (**e**). Pathology demonstrated inflammatory and fibrotic changes without malignancy, and FAP expression was confirmed in fibroblasts within the fibrotic lesions **f** The scale bars in panels **b**, **d**, and **f** represent 300 µm. FAPI, fibroblast activation protein inhibitor; PET, positron emission tomography; CT, computed tomography; FAP, fibroblast activation protein; SUVmax, maximum standardized uptake value

Among the six cases that showed significant lymph node uptake on [^18^F]FAPI PET—three with malignant involvement and three without—FAP immunohistochemical staining of the lymph node tissue demonstrated FAP expression in all nodes, regardless of malignant status. Figure 5a and b show the [^18^F]FAPI-74 PET images and FAP immunohistochemical staining of a representative case with metastatic lymph nodes. Of the three false-positives on [^18^F]FAPI-74 PET, one occurred after preoperative chemoradiation therapy, and the pathological findings of the [^18^F]FAPI-74 PET-positive lymph node revealed scar tissue without viable tumor cells (Fig. 5c and d). In the remaining two cases, [^18^F]FAPI-74 PET demonstrated uptake in multiple enlarged lymph nodes or bilateral symmetric hilar uptake suggestive of reactive lymph node swelling, and pathological findings confirmed severe inflammation and fibrosis in the affected nodes (Figs. 5e and f show a representative case).

## Discussion

In this prospective study, we performed [^18^F]FAPI-74 PET imaging and pathological evaluation of FAP expression in surgically resected primary lung cancers, including the hilar and mediastinal lymph nodes. We showed that the SUVmax of [^18^F]FAPI-74 PET correlated with the extent of FAP expression in surgically resected tumor tissues, as quantitatively evaluated by immunohistochemical staining. In addition, [^18^F]FAPI-74 PET accumulation reflected FAP expression in the hilar and mediastinal lymph nodes regardless of whether the lymph nodes had lung cancer metastasis.

FAP is preferentially expressed in CAFs; therefore, it can be used as a biomarker for identifying CAFs [3]. In a study examining CAFs in non-small cell lung cancer using single-cell RNA sequencing, FAP-positive fibroblasts were abundant in later-stage tumors (Stage ≥ Ⅱ), solid adenocarcinoma, and squamous cell carcinoma [12]. In our study, we found a significantly higher SUVmax when using [^18^F]FAPI-74 PET in non-adenocarcinoma and advanced cancer cases, which is consistent with the results of the study mentioned above [12]. However, a previous study found no significant difference in the SUVmax on FAPI-PET in primary tumors according to the histological type of lung cancer [13]. In contrast to our study, > 90% of the patients in the study by Wei et al. [13] had stage III disease, which may be the reason for the discrepancy observed between their results and those of our study. In our comparison of tracer uptake in primary tumors between [^18^F]FAPI-74 PET and [^18^F]FDG PET, adenocarcinomas exhibited a significantly higher uptake on [^18^F]FAPI-74 PET. These results indicated that FAPI-PET may be advantageous for detecting tumors with relatively low levels of tracer accumulation, such as early-stage lung adenocarcinomas. To our knowledge, few studies have quantitatively evaluated FAP immunohistochemistry in resected lung cancer specimens using an image analysis software. FAP immunohistochemistry quantified using QuPath in a prospective surgical cohort revealed that fibroblast and plasma cell positivity correlated with [^68^Ga]FAPI-46 PET uptake, underscoring the biological validity of stromal FAP expression as a determinant of tracer accumulation [14]. Our present results also demonstrate that even with different radionuclides and FAPI, FAP expression in lung cancer specimens quantitatively assessed using image analysis software correlates with FAPI-PET uptake. The present report strengthens the evidence that FAPI-PET uptake correlates with the extent of FAP expression in primary lung cancer, even in early-stage disease.

Regarding the diagnostic value of lymph node metastasis in primary lung cancer, FAPI-PET has been reported to have higher diagnostic performance for lymph node metastasis than FDG-PET [15]. In a prospective cohort of patients with stage I–IIIA non-small cell lung cancer undergoing surgical dissection, [^18^F]FAPI-42 provided higher specificity than FDG for nodal metastasis; moreover, integrating [^18^F]FAPI-42 with FDG yielded the highest N stage accuracy (83%) and prompted management revisions in more than half of the patients analyzed for clinical decision changes [16]. In the present study, the diagnostic performance of [^18^F]FAPI-74 PET for lymph node metastasis was comparable to that of FDG-PET. Furthermore, we demonstrated FAP expression around cancer cell nests in metastatic lymph nodes using immunohistochemical staining.

Notably, there were three cases without malignant findings despite significant uptake on [^18^F]FAPI-74 PET. In one case, the patient had undergone preoperative chemoradiotherapy, and the lymph node findings were compatible with treatment-related scarring; in the remaining two cases, inflammatory cells were present in the lymph nodes. A previous review of non-oncological FAPI-PET uptake reported that inflammatory reactions in the lung tissues of patients with infectious or interstitial pneumonia can lead to non-oncological tracer accumulation [17]. FAP expression in activated fibroblasts is not exclusive to malignant conditions; it is also observed in inflammatory and fibrotic conditions, where these cells contribute to extracellular matrix production, degradation, and turnover, thereby providing a biological basis for FAPI uptake in non-malignant lesions [18]. Although CAFs are generally distinguished from activated fibroblasts due to their localization within the tumor microenvironment and their roles in tumor progression and immune regulation, this distinction remains challenging because both populations are heterogeneous and share overlapping features [2]. In our study, FAP expression and corresponding [^18^F]FAPI-74 PET uptake were observed in lymph nodes with inflammation and fibrosis. These two patients also exhibited PET/CT findings suggestive of reactive uptake, such as bilateral symmetric accumulation or multiple enlarged lymph nodes, which may serve as helpful imaging clues for distinguishing inflammation-related FAPI-PET uptake from nodal metastasis.

Attention should also be paid to tissue alterations following chemotherapy and/or radiotherapy for cancer. Increased [^68^Ga]-FAPI uptake has been reported in areas of inflammation and fibrosis following radiotherapy in breast cancer, highlighting the potential for post-treatment changes to cause false-positive findings and the need for cautious image interpretation [19]. Although no previous studies have reported the correlation between FAPI-PET and histopathological evaluation of lymph nodes in lung cancer after preoperative chemoradiation, our findings indicate that histologically proven FAP expression in the hilar and mediastinal lymph nodes, together with the imaging results of FAPI-PET, suggest that treatment-related scarring and inflammatory reactions in these nodes may induce FAP expression in activated fibroblasts and thus be detected by FAPI-PET. The integration of FDG and FAPI-PET substantially reduced inflammatory false positives and overstaging, underscoring the complementary role of FDG and FAPI in improving nodal assessment accuracy in lung cancer [16, 20]. Although FAPI-PET is currently more likely to be used as a complementary tool or in specific clinical settings rather than replace FDG-PET, further efforts are warranted to distinguish non-cancer-related inflammation or therapy-induced inflammatory changes from metastasis in the hilar and mediastinal lymph nodes in lung cancer. Because both [^18^F]FAPI-74 PET and FDG-PET use ^18^F-labeled tracers, same-day imaging is impractical due to radioactive decay considerations. Additionally, FAPI-PET has not yet received global regulatory approval; further work is needed to define appropriate indications for its use in a cost-conscious clinical setting.

This study has certain limitations. First, this was a prospective study conducted at a single institution with a relatively small sample size. In particular, the number of patients with lymph node metastasis was limited, resulting in uncertainty in the estimation of nodal diagnostic performance. Second, there is an inherent selection bias. The study cohort included only patients undergoing surgical treatment, and direct comparisons were made only with those who had undergone FDG-PET, both of which should be considered potential sources of bias.

In conclusion, in resectable early-stage lung cancer, [^18^F]FAPI-74 PET correlated with histological FAP expression and proved useful for detecting lymph node metastases, although caution is warranted when interpreting the findings after preoperative therapy.

## Data Availability

All relevant data are presented in the manuscript.
